# TPRO-NET: an EEG-based emotion recognition method reflecting subtle changes in emotion

**DOI:** 10.1038/s41598-024-62990-4

**Published:** 2024-06-12

**Authors:** Xinyi Zhang, Xiankai Cheng, Hui Liu

**Affiliations:** 1 School of Biomedical Engineering (Suzhou), Division of Life Sciences and Medicine, University of Science and Technology of China, Hefei, 230026 China; 2 Suzhou Institute of Biomedical Engineering and Technology, China Academy of Science, Suzhou, 215163 China; 3 Cognitive Systems Lab, University of Bremen, Bremen, Germany

**Keywords:** Electroencephalogram, Emotion recognition, Transformer, Convolutional neural network, Minuscule emotional changes, Biomedical engineering, Computer science, Scientific data

## Abstract

Emotion recognition based on Electroencephalogram (EEG) has been applied in various fields, including human–computer interaction and healthcare. However, for the popular Valence-Arousal-Dominance emotion model, researchers often classify the dimensions into high and low categories, which cannot reflect subtle changes in emotion. Furthermore, there are issues with the design of EEG features and the efficiency of transformer. To address these issues, we have designed TPRO-NET, a neural network that takes differential entropy and enhanced differential entropy features as input and outputs emotion categories through convolutional layers and improved transformer encoders. For our experiments, we categorized the emotions in the DEAP dataset into 8 classes and those in the DREAMER dataset into 5 classes. On the DEAP and the DREAMER datasets, TPRO-NET achieved average accuracy rates of 97.63%/97.47%/97.88% and 98.18%/98.37%/98.40%, respectively, on the Valence/Arousal/Dominance dimension for the subject-dependent experiments. Compared to other advanced methods, TPRO-NET demonstrates superior performance.

## Introduction

Emotions play a significant role in daily life, reflecting the connection between subjective needs and the objective external world^1^. Positive emotions have a beneficial impact on people's physical and mental health, while negative emotions have the opposite effect^2^. In recent years, there has been a growing interest in applications related to emotion recognition, such as human–computer interaction^3^ and psychological disease rehabilitation^4^. Due to the excellent characteristics of Electroencephalogram (EEG) signals in terms of their inability to be falsified, high time resolution, and sensitivity to emotional changes^5,6^, emotion recognition based on EEG signals has received attention from both the academic and industrial communities.

Discrete and continuous emotion models are commonly used to measure emotions^7^. Discrete emotion models divide emotions into finite categories, such as happiness, sadness, surprise, fear, anger, and disgust^8^. Continuous emotion models measure emotions using dimensions and can describe more complex emotions^7^. The VAD (Valence-Arousal-Dominance) emotion model^9^, which is currently popular, uses valence, arousal, and dominance to measure emotions. Valence corresponds to the degree of pleasure, arousal corresponds to the degree of intensity, and dominance corresponds to the degree of subjective control^7^. The lower the values of these dimensions, the lower the degree of the corresponding emotion and vice versa^7^.

Emotion recognition based on EEG signals involves two stages: feature extraction and classification algorithms. Various features for EEG signals have been proposed, including fractal dimension features^10^, high-order cross features^11^, power spectral density^12^, differential entropy (DE)^13^, Riemannian manifold features^14^, differential causality^15^, and the discrete wavelet transform^16^. Deep learning classification algorithms, such as transformer^7,17–19^, graph neural network^20,21^, and convolutional neural network^2,22^, are commonly used for emotional category classification.

Although these efforts have yielded convincing results, there are still some issues. Firstly, due to the time asymmetry, instability, and low signal-to-noise ratio of EEG signals^23^, researchers often use various complex methods to design robust features^24,25^, which may not be conducive to practical applications. Secondly, although transformer performs well on EEG-based emotion classification tasks^7,13,18,19^, it requires a large amount of computation^26,27^. Finally, in VAD emotion models, researchers typically categorize dimensions into high- and low-state labels^2,7,17,20–22^, which may not capture nuanced changes in emotion.

To address these issues, we propose TPRO-NET to recognize complex emotions. The model takes differential entropy features and enhanced differential entropy features as inputs, and outputs emotion categories through convolutional neural networks and improved transformer encoders. The main contributions of this article are as follows:

1. A Simple nonlinear transformation is proposed to enhance the robustness of differential entropy features.
2. By improving the transformer encoder, we reduce the amount of computation while ensuring the similar performance as the transformer encoder does.
3. TPRO-NET achieved state-of-the-art results by completing an 8-class emotion classification task on the DEAP dataset and a 5-class emotion classification task on the DREAMER dataset.

This article is organized as follows: Section "Related works" reviews previous work on feature extraction and transformer. Section "Materials" presents the materials used in this study. Section "Methods" describes the proposed EEG-based emotion recognition algorithm TPRO-NET. Section "Experiments results and analysis" presents the experimental results and analysis of the DREAMER and DEAP datasets. Section "Discussion" discusses the experiments. Finally, Section "Conclusion" provides a summary of the paper and discusses future work.

## Related works

Researchers have made significant progress in the field of emotional recognition based on EEG signals. Meanwhile, there are still some existing flaws and issues that require attention.

Wei, et al.^7^ proposed the transformer capsule network to address the challenges of capturing global contextual information across the temporal, frequency, intra-channel, and inter-channel domains in convolutional neural networks. Subject-dependent binary classification experiments were conducted on the DEAP (DREAMER) dataset, achieving accuracies of 98.76% (98.59%), 98.81% (98.61%), and 98.82% (98.67%) on the dimensions of Valence, Arousal and Dominance, respectively. However, the model's performance was average in cross-subject EEG emotion recognition tasks, indicating that it may not fully account for the inter-individual differences in EEG patterns, which could affect its universality and applicability.

Chang, et al.^16^ put forward an automatic transformer neural architecture search framework based on multi-objective evolutionary algorithm to avoid the time-consuming and resource-intensive process of designing neural networks. This framework achieved an accuracy rate of over 95% in subject-dependent binary classification experiments on the DEAP and the DREAMER datasets. Nevertheless, the model parameters of this method amount to 6.98M, which is not lightweight enough. Still, the model's performance in cross-subject EEG emotion recognition is average.

Yin, et al.^21^ aimed to achieve breakthroughs in model performance metrics. They employed graph neural networks to extract non-Euclidean spatial features and utilized long short-term memory to search for temporal features in order to perform binary classification of emotions. Conducting subject-dependent experiments on the DEAP dataset, they achieved an accuracy of 90.45% and 90.60% on the Valence and Arousal dimensions, respectively. In cross-subject experiments, they obtained accuracies of 84.81% and 85.27% on Valence and Arousal, respectively. It is worth pointing out that the method was validated using only the DEAP dataset, which may limit the model's effectiveness. Other limitations of this model include its inability to recognize multiple emotions, high computational complexity, and the requirement for a long-time window for data truncation that potentially hinders its real-time application.

Cheng, et al.^2^ proposed a random convolutional neural network to reduce the computational complexity of the backpropagation process, inspired by the successes of random vector functional link and convolutional random vector functional link. Binary classification experiments were conducted on the DEAP dataset, demonstrating that the model achieved accuracies of over 99% on the dimensions of valence and arousal. Being validated by only one dataset cannot demonstrate its robustness in different contexts.

Choo, et al.^28^ investigated the effectiveness of multi-task learning for emotion recognition using raw EEG-based convolutional neural networks (CNNs) with auxiliary context information. The study utilized temporal and spatial filtering layers from raw EEG-based CNNs as shared and task-specific layers for emotion and context classification tasks. The experiments were conducted using the authors’ own dataset, on which promising results were achieved. Noteworthy, their dataset only includes negative emotions, and the proposed model has limited generalization ability.

Due to the nonlinearity and non-stationarity of EEG signals, extracting effective non-stationary and valid features can be challenging. To address this issue, Zhong, et al.^24^ developed a new feature extraction method called tunable Q-factor wavelet transform (TQWT), a spatiotemporal representation method for multichannel EEG signals, and a hybrid convolutional recurrent neural network. The model achieved an accuracy of 95.33% on the SEED dataset. Besides only tested by one dataset, the bottlenecks also include the complex computation of TQWT, which requires numerous floating-point operations.

Liu, et al.^29^ focused on enhancing the performance of the model, proposing an EEG emotion recognition model based on the attention mechanism and a pre-trained convolution capsule network to recognize various emotions more effectively. This model utilizes coordinate attention to endow the input signal with relative spatial information, and subsequently maps the EEG signal to a higher dimensional space, thereby enriching the emotion-related information within the EEG. They conducted experiments using only the DEAP dataset, achieving good performance in subject-independent experiments and moderate performance in subject-dependent experiments. Still, only one dataset was applied for the model validation, and cross-subject results are not ideal.

Lin, et al^30^ proposed the dual-scale EEG-Mixer to address the limitation of CNNs in extracting global information. This model fuses spatial and frequency domain features of EEG signals, achieving an accuracy of over 95% on the DEAP dataset and 93.69% on the SEED dataset. The model, nonetheless, does not effectively utilize the temporal information of EEG signals and performs moderately in cross-subject experiments. This work demonstrates experimentally that transformers require significant computational resources.

Tang, et al.^31^ proposed the spatial-temporal information learning network to extract discriminative features from EEG signals. The network captures spatial correlations and temporal contexts using a combination of a convolutional neural network, convolutional block attention module, and bidirectional long short-term memory. Subject-independent experiments were conducted using only one dataset, and moderate results were achieved.

Several shortcomings have been identified in previous works, including validating model effectiveness using only one dataset, difficulty in cross-subject emotion recognition from EEG signals, challenges in incorporating temporal, spatial, and frequency domain features into EEG signal characteristics simultaneously, high computational complexity in feature extraction, low computational efficiency of the transformer model, and a limited number of emotional categories recognized by the VAD model. In addition to the literature described in detail above, there are other works in EEG fields that have also mentioned these issues, such as^32–34^.

Standing on the shoulders of predecessors’ work, this article focuses on improving model performance by utilizing simplified features, enhancing the efficiency of the transformer, and developing algorithms to recognize subtle changes in emotions. Due to this, we have designed TPRO-NET, a neural network that takes differential entropy and enhanced differential entropy features as input and outputs emotion categories through convolutional layers and improved transformer encoders.

## Materials

The open-source DEAP^35^ and the DREAMER^36^ datasets are commonly used for EEG-based emotion recognition. Both datasets induce emotion-related EEG signals through video stimuli and determine emotion labels through subjective scoring by participants.

**Figure 1:**
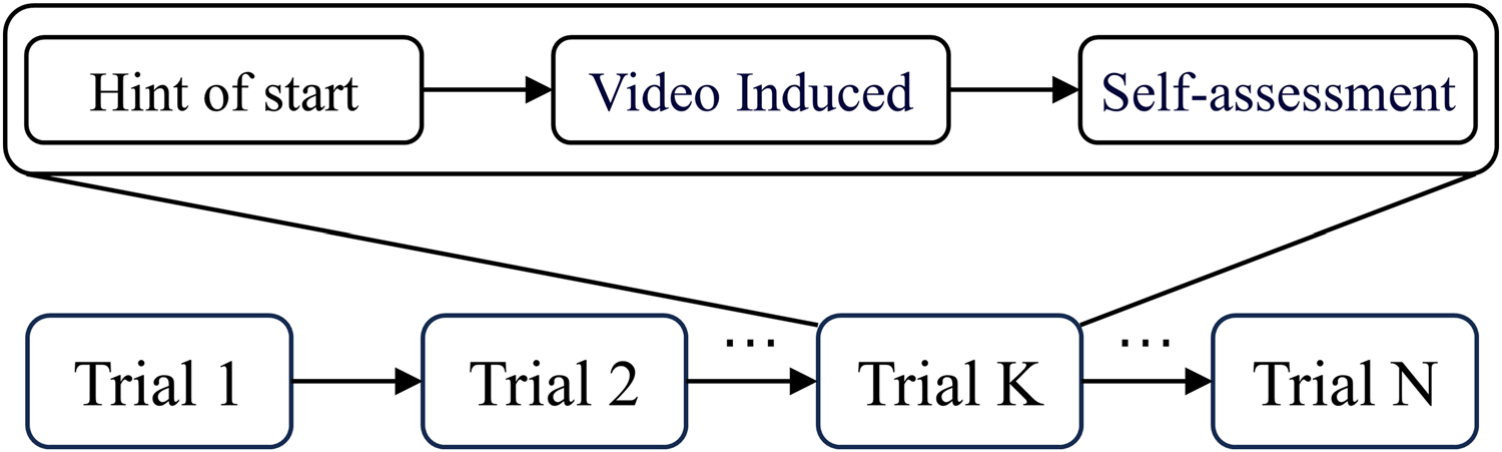
General steps of video-induced emotion.

The DEAP dataset includes 40 videos that induce emotions in 32 subjects and collects EEG signals from 32 channels. For each video stimulus, participants must provide a floating-point score ranging from 1 to 9 for the four dimensions of valence, arousal, dominance, and liking to measure their emotions. Each elicitation produces 3 s of baseline signals and 60 s of experimental signals, resulting in a total of 2520 ((60 + 3) × 40) seconds of EEG signals per subject. The DEAP dataset provides two types of signals: the original signals with a sample rate of 512 Hz and the signals that have been downsampled to 128 Hz and have undergone independent component analysis to remove EOG artifacts. We conducted experiments using the processed signals with experimental signals. The general scheme of video-induced emotion is depicted in Fig. 1.

The DREAMER dataset includes 18 videos that induce emotions in 23 subjects and collects EEG signals from 14 channels. For each video stimulus, the participant was asked to provide an integer score ranging from 1 to 5 on three dimensions: valence, arousal, and dominance, to measure their emotions. The length of each elicitation video ranges from 65 to 393 s. The Dreamer dataset provides signals that have been downsampled to 128 Hz and have undergone eye artifact removal using a linear phase finite impulse response filter. The general scheme of video-induced emotion is depicted in Fig. 1.

## Methods

**Figure 2:**
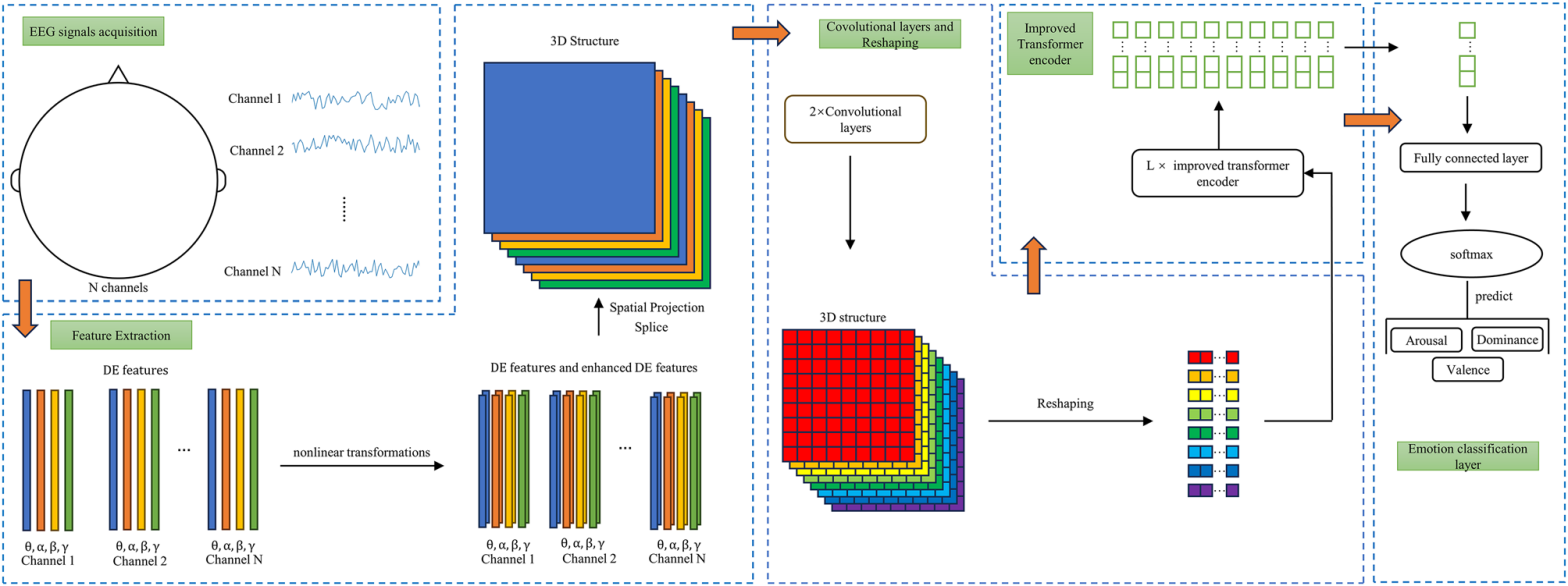
Detailed structure of TPRO-NET.

The proposed EEG emotion recognition algorithm, TPRO-NET, comprises four parts: feature extraction, convolutional layers and reshaping, improved transformer encoder, and emotion classification layer. Figure 2 illustrates the detailed structure of TPRO-NET.

### Feature extraction

The processed EEG signals from the datasets mentioned in Section "Materials" are segmented into non-overlapping one-second segments. Immediately following that, we apply a fourth-order band-pass Butterworth filter to each of the four frequency bands ($\theta (4-8Hz),\alpha (8-13Hz),\beta (13-31Hz),\gamma (31-45Hz$))^37,38^ to obtain the filtered signals. To improve computational efficiency, the differential entropy feature can be approximated as a Gaussian distribution between 4 and 45 Hz^11^. The calculation formula for this approximation is as follows.

1 $$\begin{array}{*{20}c} {\text{DE}\left( X \right) = \frac{1}{2}\log \left( {2\pi \text{e}\sigma^{2} } \right)} \\ \end{array}$$

where *X* represents a one-second EEG signal segment, σ represents the standard deviation of the Gaussian distribution, and $\uppi$, $\text{e}$ are constants.

To improve feature robustness, we normalize the differential entropy features calculated for each channel using the following formula:

2 $$\begin{array}{*{20}c} {x_{i} = \frac{{x_{i} - \min \left( {x_{i} } \right)}}{{\max \left( {x_{i} } \right) - \min \left( {x_{i} } \right)}} \quad i = 1,2, \ldots ,m} \\ \end{array}$$

where $x_{i}$ represents the value of the differential entropy feature within a channel, and m represents the total number of differential entropy features in a channel.

**Figure 3:**
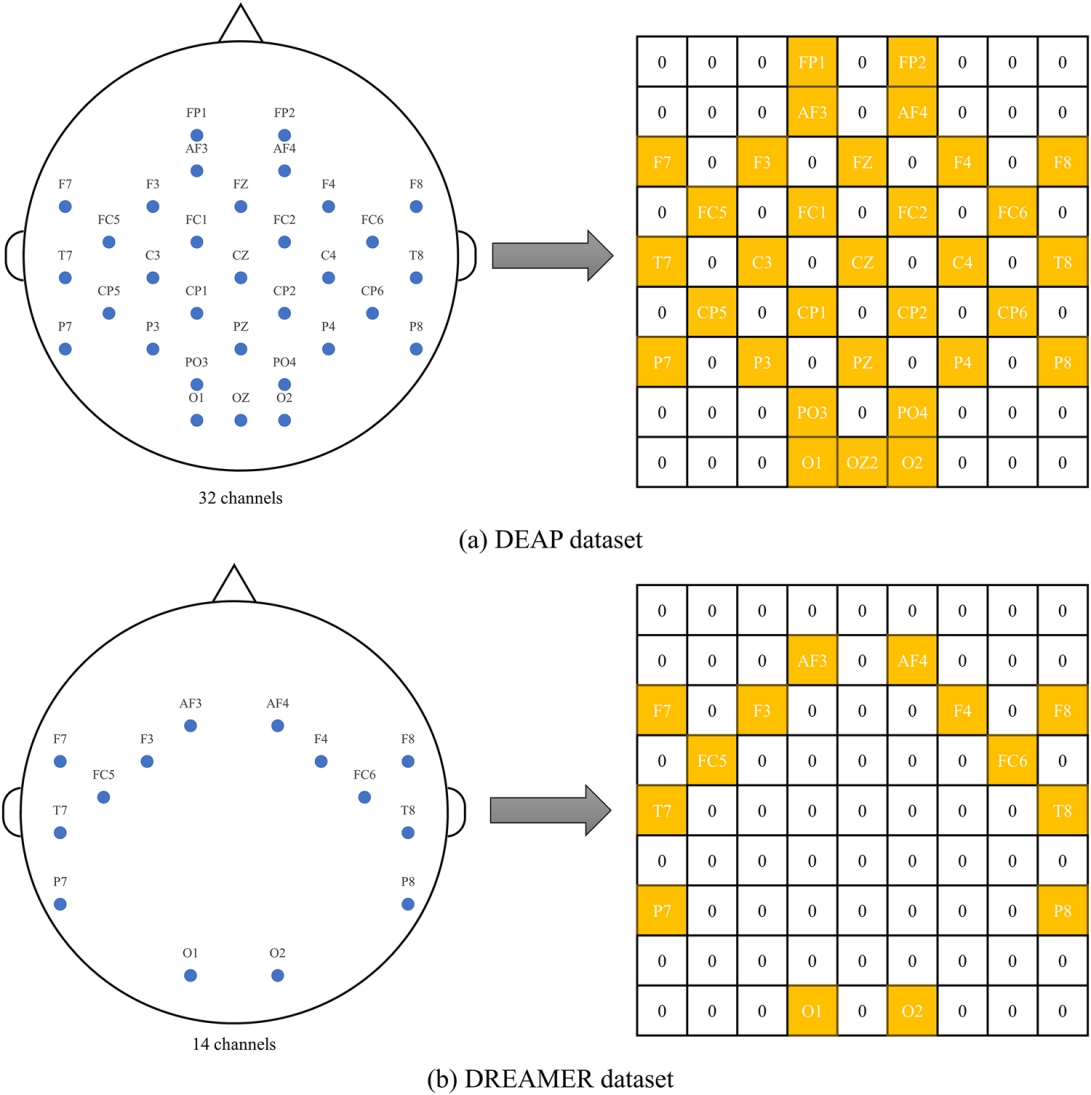
Detailed process of spatial mapping.

Similarly to previous studies^22,39^, we map the differential entropy feature to a two-dimensional matrix with dimensions of $H\times W (H=9, W=9)$ based on the spatial location of the channel to include spatial information. The values in the locations that are not mapped are set to 0 by default. The detailed spatial mapping method is shown in Fig. 3. Currently, a one-second EEG signal segment can produce four two-dimensional matrices based on the four different frequency bands ($\theta , \alpha , \beta , \gamma$).

To obtain more robust features, we applied nonlinear transformation to the four two-dimensional matrices we calculated. The calculation formula for nonlinear transformation is as follows.

3 $$\begin{array}{*{20}c} {M_{1} = \log \left( {M + 1} \right)} \\ \end{array}$$

where *M* represents the matrix after spatial mapping, and ${M}_{1}$ represents the matrix after nonlinear transformation. In addition, to improve the calculation accuracy of single precision floating point numbers, we multiplied the coefficient by 255. Through this step, four differential entropy feature matrices and four enhanced differential entropy feature matrices can be obtained. These two-dimensional matrices are organized into a three-dimensional structure with a size of $C\times H\times W$ to obtain the input of the neural network, where $C=8$ represents the number of matrices.

### Convolutional layers and reshaping

By feeding the features obtained in 4.1 into convolutional layers to learn global features, tensor $x\in {R}^{C\times H\times W}$ can be obtained. Table 1 shows the hyperparameters used in the convolutional layers. Afterwards, in order to match the input of the improved transformer encoder, we reshape *x* using formula 4.

4 $$\begin{array}{*{20}c} {z_{0} = \text{Reshape}\left( x \right)} \\ \end{array}$$

where $z_{0} \in R^{{C \times \left( {H \times W} \right)}}$ represents the input of improved transformer encoder.

**Table 1:** Hyperparameters of two convolutional layers.

Hyperparameters	1st convolutional layer	2nd convolutional layer
Kernel size	3	3
Stride	1	1
Padding	1	1
Input channels	8	32
Output channels	32	8

### Improved transformer encoder

**Figure 4:**
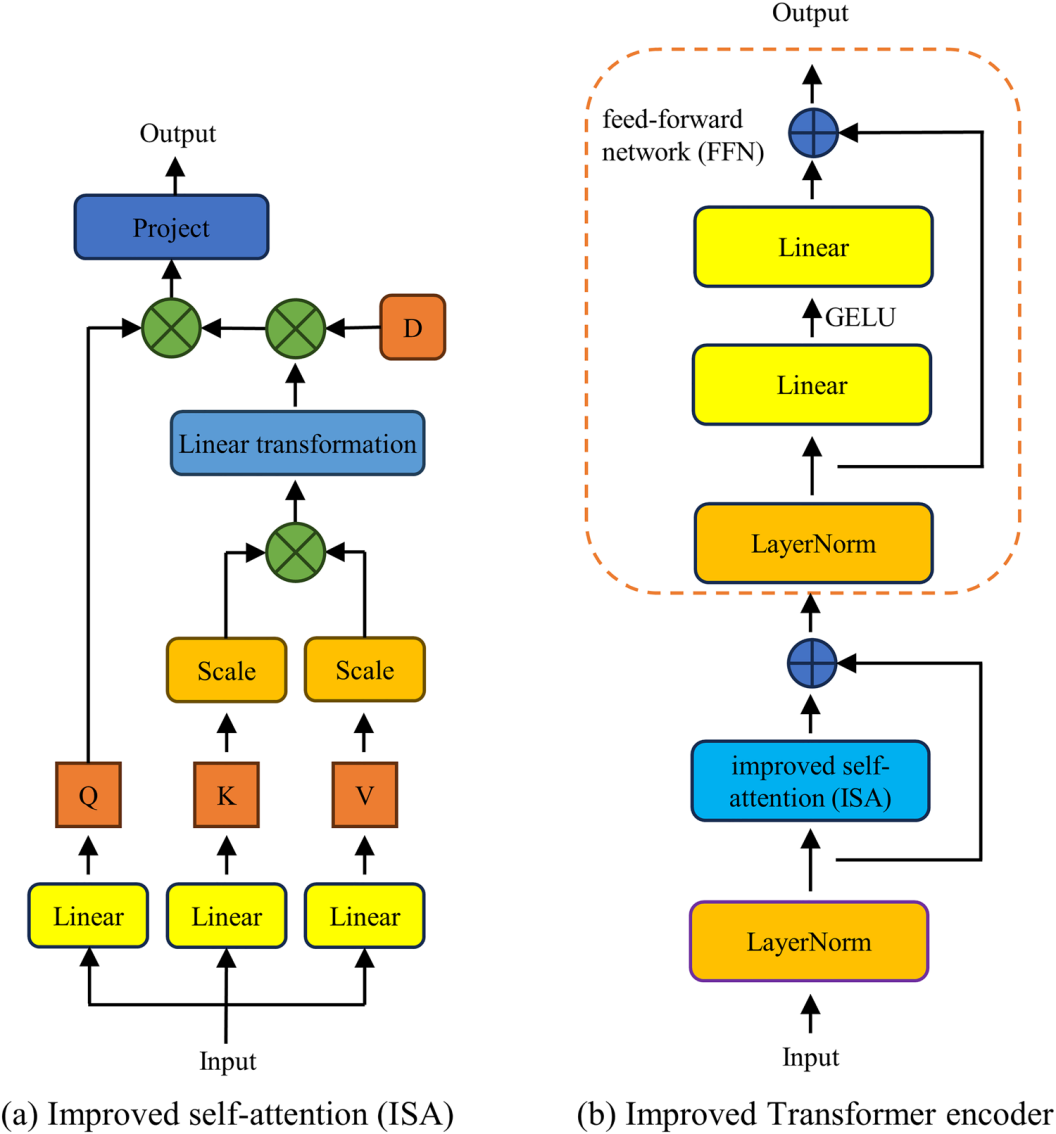
Detailed structure of improved transformer.

The improved transformer encoder mainly consists of two parts: the attention mechanism ISA and the feedforward neural network (FFN). The structure of the improved transformer is presented in Fig. 4. Attention mechanism ISA first calculates Q, K, and V using the method provided in^40^, and then learns local features. The relevant calculation formulas are shown below.

5 $$\begin{array}{*{20}c} {\left[ {Q,K,V} \right] = z_{0} U_{qkv} } \\ \end{array}$$

6 $$\begin{array}{*{20}c} {\text{ISA}\left( {Q,K,V} \right) = U_{proj} \left( {D \odot Q \odot \left( {U_{copy} \left( {\frac{K}{{Scale_{K} }} \odot \frac{V}{{Scale_{V} }} } \right)U_{sum} } \right)} \right)} \\ \end{array}$$

7 $$\begin{array}{*{20}c} {Scale_{V} = \left| {\left| {V_{ - 1} } \right|} \right|_{2} \quad Scale_{Q} = \left| {\left| {Q_{ - 1} } \right|} \right|_{2} } \\ \end{array}$$

where *Q*, *K* and *V* represent queries, keys, and values^40^, respectively, *D* represents a trainable dynamic scaling matrix with an initial value of 1, ${U}_{sum}, {U}_{copy}$, ${U}_{qkv},{U}_{proj}$ represent linear transformation matrices, $Scal{e}_{K}, Scal{e}_{V}$ represent the L2 norms of the last dimension of keys and values, respectively, and ‘$\odot$’ represents the operation of the Hadamard product.

The FFN is composed of four components: layer normalization (LN), linear layer (Linear), GELU activation function, and residual connection structure. The relevant calculation formulas are presented below.

8 $$\begin{array}{*{20}c} {z_{l}^{\prime } = {\text{ISA}}\left( {{\text{LN}}\left( {z_{l - 1} } \right)} \right) + z_{l - 1} \quad l = 1,2, \ldots ,L} \\ \end{array}$$

9 $$\begin{array}{*{20}c} {z_{l} = {\text{Linear}}\left( {\text{GELU}\left( {\text{Linear}\left( {\text{LN}\left( {z_{l}^{\prime } } \right)} \right)} \right)} \right) + \text{LN}\left(z_{l}^{\prime }\right) l = 1,2, \ldots ,L} \\ \end{array}$$

where ${z}_{l}{\prime},{z}_{l}\in {R}^{\left(C+2\right)\times \left(H\times W\right)}$ represent the intermediate variables and the output of the $lth$ improved transformer encoder, respectively, and $L=15$ represents the number of sequentially connected improved transformer encoders.

### Classification layers

For the tensor ${z}_{L}\in {R}^{\left(C+2\right)\times \left(H\times W\right)}$ outputed by the improved transformer encoder in series, we perform linear transformation to obtain ${z}_{D}\in {R}^{\left(H\times W\right)}$, and then pass it through a fully connected layer and a SoftMax layer to obtain the final classification result. Figure 2 shows the detailed process.

## Experiments results and analysis

### Experiment setup

The performance of TPRO-NET was tested on a NVIDIA RTX 4090 using the DEAP dataset^35^ and the DREAMER dataset^36^ with the PyTorch framework. The batch size was set to 240, the learning rate was set to 0.001, and the optimizer was AdamW. Additionally, the number of epochs was set to 100 for the DEAP dataset and 50 for the DREAMER dataset. We conducted subject-dependent experiments, where training data and testing data from the same subjects, with five-fold cross-validation, as in many past works^1,7,31,39,41–45^.

Two types of subject-dependent experiments, where training data and testing data from the same subjects, with five-fold cross-validation^1,7,31,39,41–45^ were conducted: binary classification and multi-classification. In the first type of subject-dependent experiment, the labels were divided into high and low categories. A threshold of 5 was used for the DEAP dataset (low: < 5, high: $\ge$5) and a threshold of 3 was used for the DREAMER dataset (low: < 3, high: $\ge$3). The strings ‘lv’, ‘la’, ‘ld’, ‘hv’, ‘ha’ and ‘hd’ represent low valence, low arousal, low dominance, high valence, high arousal and high dominance, respectively. In the second type of experiment, the labels are divided into as many categories as possible. For the DEAP dataset, floating-point labels in the range [1,9] are equally divided into 8 parts to obtain 8 categories. The characters '1', '2', '3', '4', '5', '6', '7', '8' represent the eight intervals [1,2], (2,3], (3,4], (4,5], (5,6], (6,7], (7,8], (8,9] respectively. For the DREAMER dataset, the 5 categories provided by the dataset are directly used. The characters '1', '2', '3', '4', '5' represent the integer labels 1, 2, 3, 4, 5, respectively. Tables 2 and 3 show the sample size of each category for binary classification and multi-class classification in the DEAP and the DREAMER datasets.

**Table 2:** The number of samples in each category of the DEAP dataset.

DEAP	Multiple classes								Binary classes	
	1	2	3	4	5	6	7	8	l	h
Valence	6780	6540	8700	12,300	10,140	10,680	13,200	8460	33,360	43,440
Arousal	6600	6960	9000	10,020	12,780	13,860	11,760	5820	31,560	45,240
Dominance	5220	6600	8880	9300	14,340	11,340	10,380	10,740	29,100	47,700

**Table 3:** The number of samples in each category of the DREAMER dataset.

DREAMER	Multiple classes					Binary classes	
	1	2	3	4	5	l	h
Valence	17,176	16,338	16,979	21,118	14,116	50,510	35,234
Arousal	3505	16,431	24,719	30,026	11,063	44,655	41,089
Dominance	2279	14,948	22,470	30,816	15,231	39,697	46,047

We use macro average of accuracy, precision, specificity, recall, and F1-score due to the imbalanced sample distribution to evaluate the model's performance. The formulas of accuracy, precision, specificity, recall and F1-score with parameters of true positives (TP), false positives (FP), true negatives (TN), false negatives (FN) are as follows:

10 $$\begin{array}{*{20}c} {\text{Accuracy} = \frac{{{\text{TP}} + {\text{TN}}}}{{{\text{TP}} + {\text{FP}} + {\text{TN}} + {\text{FN}}}}} \\ \end{array}$$

11 $$\begin{array}{*{20}c} {{\text{Precision}} = \frac{{{\text{TP}}}}{{{\text{TP}} + {\text{FP}}}}} \\ \end{array} { }$$

12 $$\begin{array}{*{20}c} {{\text{Specificity}} = \frac{{{\text{TN}}}}{{{\text{TN}} + {\text{FP}}}}} \\ \end{array}$$

13 $$\begin{array}{*{20}c} {{\text{Recall}} = \frac{{{\text{TP}}}}{{{\text{TP}} + {\text{FN}}}}} \\ \end{array}$$

14 $$\begin{array}{*{20}c} {{\text{F1 - score}} = 2 \times \frac{{{\text{Precision}} \times {\text{Recall}}}}{{{\text{Precision}} + {\text{Recall}}}}} \\ \end{array}$$

### Subject-dependent experiments

**Figure 5:**
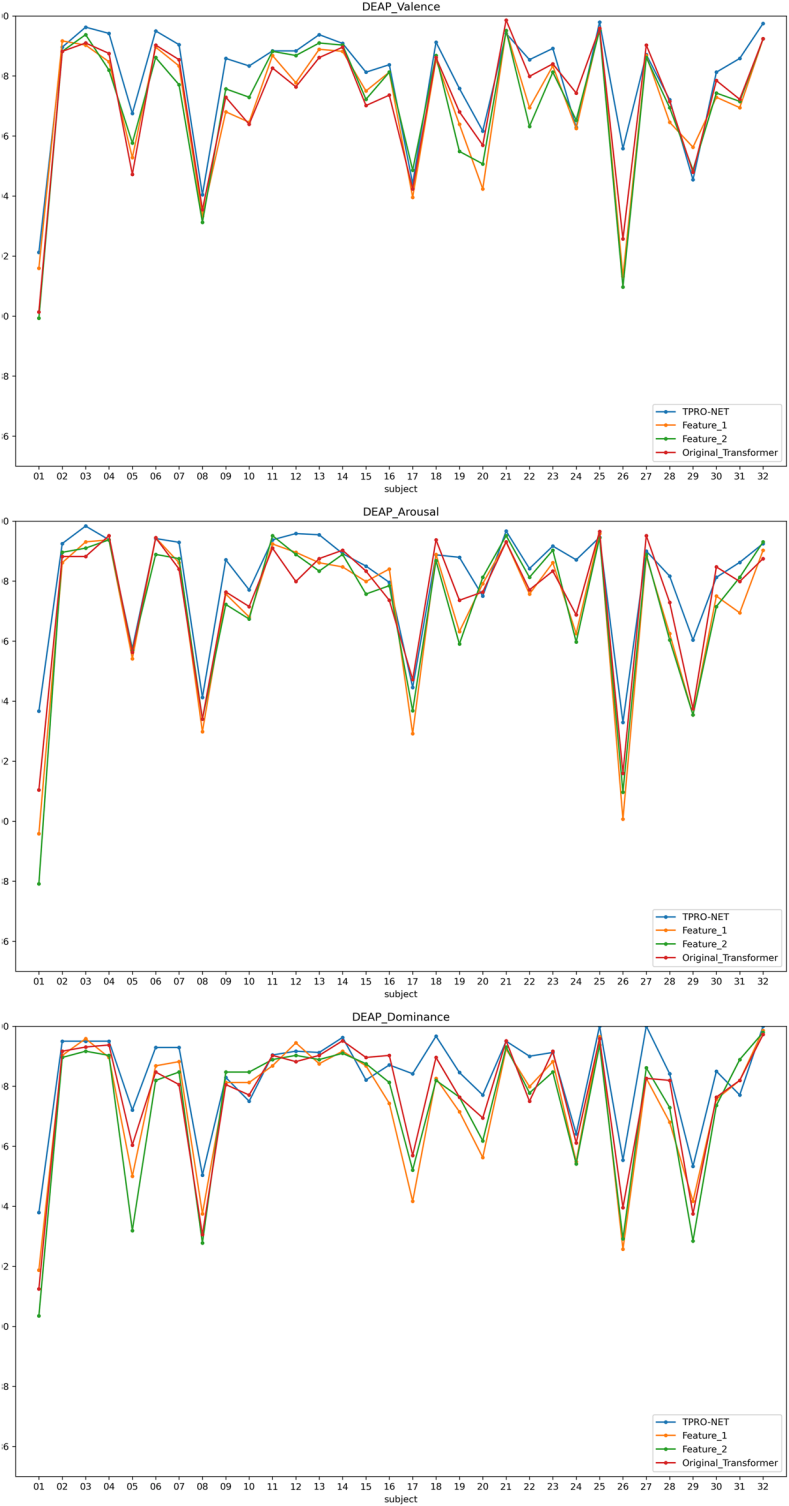
The results of the subject-dependent experiment with binary classes on the Valence (top), Arousal (middle), and Dominance (bottom) dimensions of DEAP.

**Figure 6:**
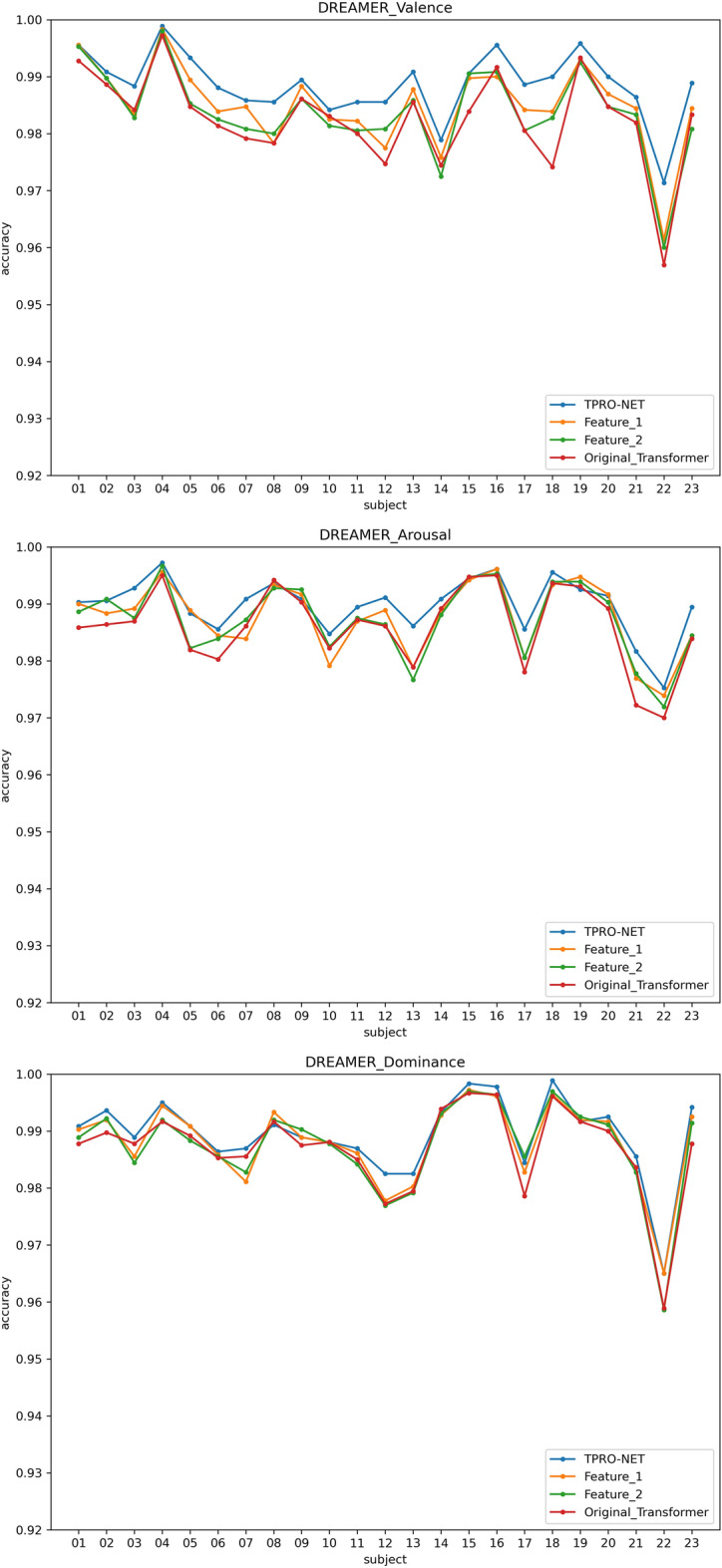
The results of the subject-dependent experiment with binary classes on the Valence (top), Arousal (middle), and Dominance (bottom) dimensions of DREAMER.

**Figure 7:**
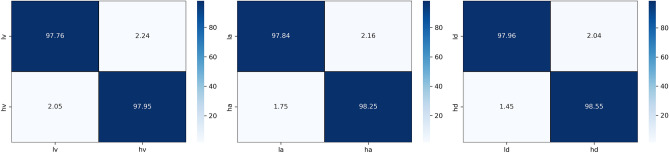
Confusion matrices for the subject-dependent binary-class experiments on the DEAP dataset.

**Figure 8:**
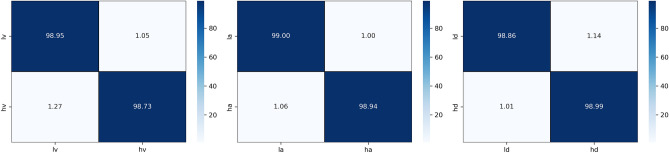
Confusion matrices for the subject-dependent binary-class experiments on the DREAMER dataset.

**Figure 9:**
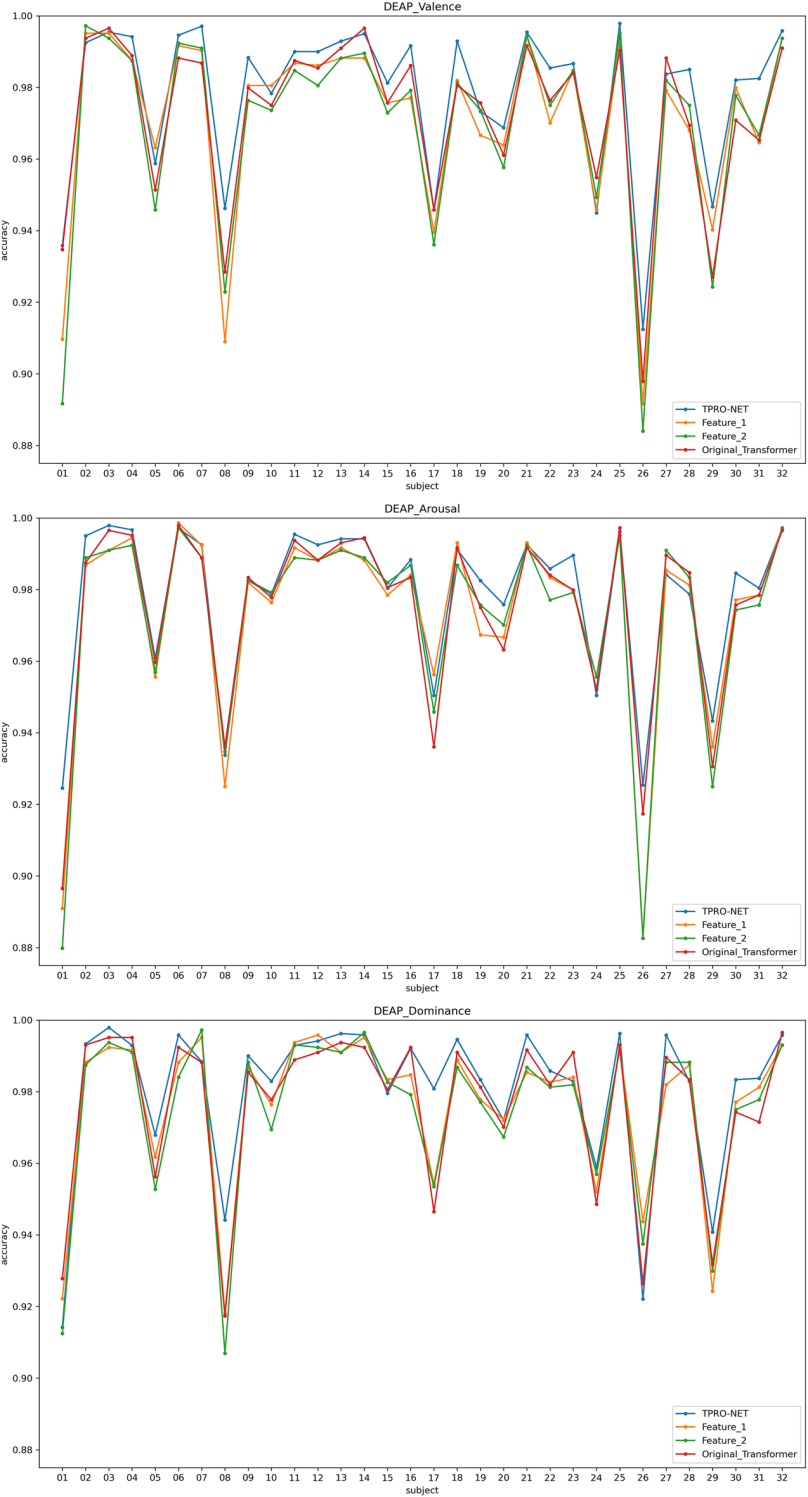
The results of the subject-dependent experiment with multiple classes on the Valence (top), Arousal (middle), and Dominance (bottom) dimensions of DEAP.

**Figure 10:**
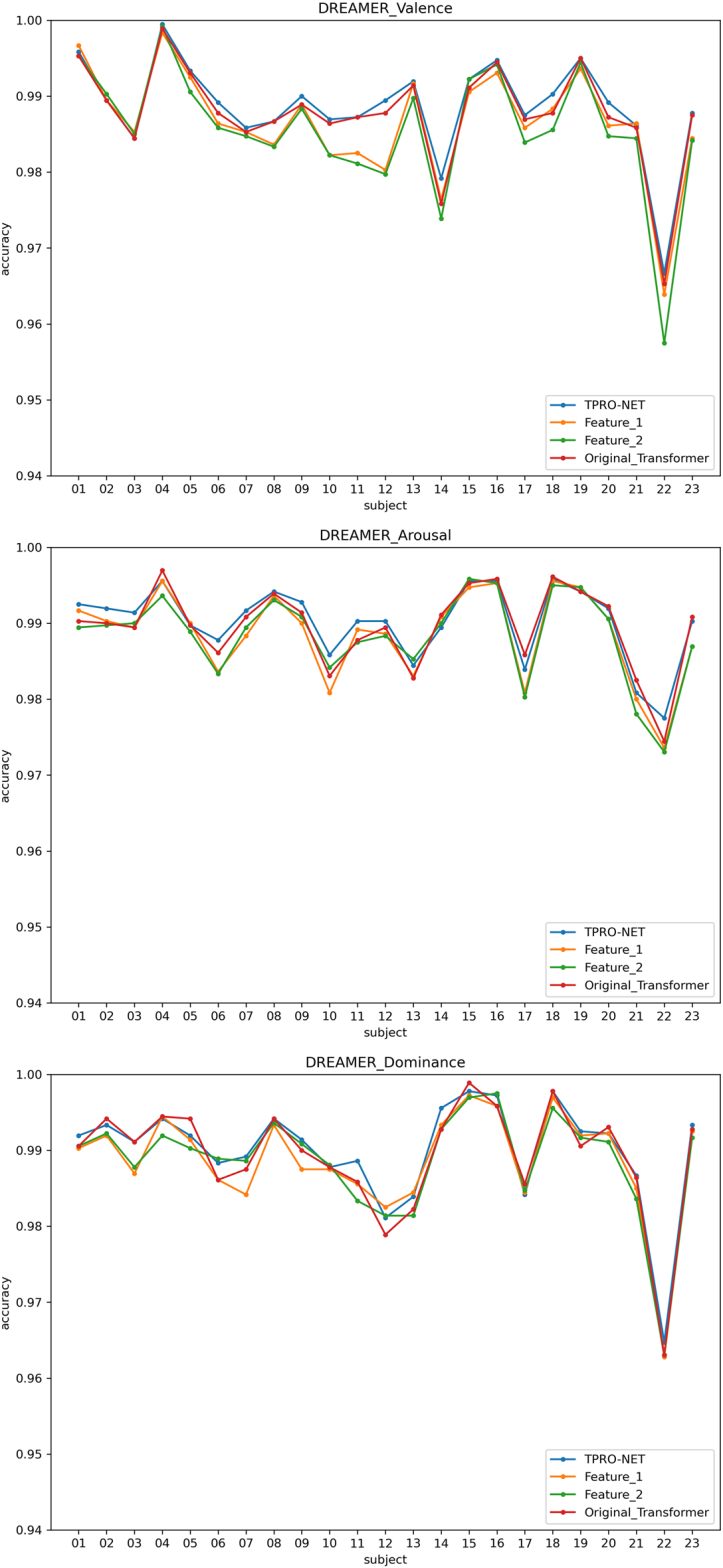
The results of the subject-dependent experiment with multiple classes on the Valence (top), Arousal (middle), and Dominance (bottom) dimensions of DREAMER.

**Figure 11:**
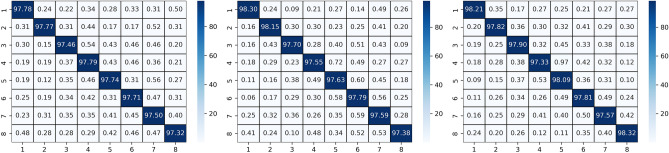
Confusion matrices for the multiple-class subject-dependent experiments on the DEAP dataset.

**Figure 12:**
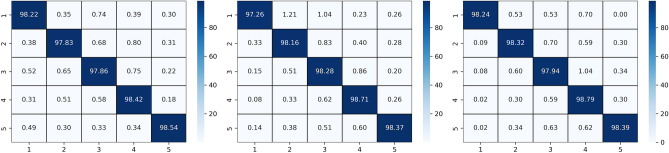
Confusion matrices for the multiple-class subject-dependent experiments on the DREAMER dataset.

Two types of subject-dependent experiments were conducted: binary classification and multi-classification. Table 4 lists the average experimental outcomes of TPRO-NET on the DEAP and the DREAMER datasets. The binary classification results of TPRO-NET on the DEAP and the DREAMER datasets for each subject are shown in Figs. 5 and 6, respectively, by the blue lines. Similarly, the multiple classification results of TPRO-NET on the DEAP and the DREAMER datasets for each subject are shown in Figs. 7 and 8, respectively, by the blue lines. The confusion matrices presented in Figs. 9, 10, 11, and 12 demonstrate that TPRO-NET has exceptional discrimination capabilities for each category in both two-class and multi-class classifications on the DEAP and the DREAMER datasets.

**Table 4:** The results of the subject-dependent experiments ($\text{mean}\pm \text{standard deviation}$).

	DEAP			DREAMER		
	Valence	Arousal	Dominance	Valence	Arousal	Dominance
TPRO-NET (2 classes)						
Accuracy	$97.87\pm 1.89$	$98.08\pm 1.83$	$98.33\pm 1.55$	$98.86\pm 0.57$	$98.97\pm 0.49$	$98.93\pm 0.69$
Precision	$97.73+1.92$	$97.82\pm 1.95$	$98.02\pm 1.87$	$98.76\pm 0.67$	$98.83\pm 0.56$	$98.81\pm 0.76$
Specificity	$97.73+1.92$	$97.82\pm 1.95$	$98.02\pm 1.87$	$98.76\pm 0.67$	$98.83\pm 0.56$	$98.81\pm 0.76$
Recall	$97.82\pm 1.90$	$97.96\pm 1.86$	$98.20\pm 1.63$	$98.82\pm 0.57$	$98.85\pm 0.55$	$98.84\pm 0.72$
F1-score	$97.78\pm 1.91$	$97.89\pm 1.90$	$98.11\pm 1.74$	$98.79\pm 0.62$	$98.84\pm 0.55$	$98.82\pm 0.74$
TPRO-NET (multiple classes)						
Accuracy	$97.63\pm 2.38$	$97.74\pm 2.26$	$97.88\pm 2.24$	$98.18\pm 0.97$	$98.37\pm 0.93$	$98.40\pm 0.80$
Precision	$97.51\pm 2.34$	$97.65\pm 2.28$	$97.61\pm 2.50$	$98.06\pm 1.03$	$98.03\pm 1.13$	$98.10\pm 1.15$
Specificity	$99.60\pm 0.41$	$99.60\pm 0.40$	$99.49\pm 0.68$	$99.51\pm 0.26$	$99.44\pm 0.32$	$99.42\pm 0.31$
Recall	$97.85\pm 2.09$	$97.86\pm 2.15$	$97.86\pm 2.24$	$98.08\pm 1.02$	$98.22\pm 1.05$	$98.18\pm 0.90$
F1-score	$97.67\pm 2.22$	$97.75\pm 2.22$	$97.73\pm 2.35$	$98.07\pm 1.02$	$98.12\pm 1.08$	$98.14\pm 1.03$

The experimental results demonstrate that TPRO-NET effectively matches the characteristics of EEG signals to accomplish both multi-classification tasks that reflect subtle changes in emotion and conventional binary classification tasks.

### Influence of different types of features on the results

In addition to DE features, we also use a nonlinear transformation as shown in Equation 4 to enhance DE features for better results. To demonstrate the effectiveness of the enhanced DE features, we conducted subject-dependent experiments using the DE features and the enhanced DE features, respectively. In the relevant tables of the experimental results, the expressions"Feature_1" and "Feature_2" represent the DE feature and the enhanced DE feature, respectively.

The results are shown in Table 5, as well as the orange and green lines of Figs. 5, 6, 7, and 8, indicating that there is no significant performance gap between the DE features and the enhanced DE features, and that there is a complementary relationship between them. By comparing the experimental results in Section "Subject-dependent experiments", using both DE features and enhanced DE features can improve the performance of TPRO-NET in most cases.

**Table 5:** The results of subject-dependent experiments on different features. ($\text{mean}\pm \text{standard deviation}$).

Category	DEAP			DREAMER		
	Valence	Arousal	Dominance	Valence	Arousal	Dominance
Feature_1 (2 classes)						
Accuracy	$96.49\pm 2.70$	$97.46\pm 2.27$	$97.86\pm 2.02$	$98.50\pm 0.73$	$98.75\pm 0.62$	$98.80\pm 0.72$
Precision	$96.27\pm 2.75$	$97.14\pm 2.37$	$97.46\pm 2.27$	$98.34\pm 0.87$	$98.62\pm 0.67$	$98.67\pm 0.78$
Specificity	$96.27\pm 2.75$	$97.14\pm 2.37$	$97.46\pm 2.27$	$98.34\pm 0.87$	$98.62\pm 0.67$	$98.67\pm 0.78$
Recall	$96.39\pm 2.73$	$97.28\pm 2.33$	$97.70\pm 2.14$	$98.47\pm 0.73$	$98.59\pm 0.69$	$98.70\pm 0.75$
F1-score	$96.33\pm 2.74$	$97.20\pm 2.34$	$97.58\pm 2.19$	$98.40\pm 0.80$	$98.61\pm 0.67$	$98.68\pm 0.76$
Feature_1 (multiple classes)						
Accuracy	$96.35\pm 3.29$	$97.14\pm 2.75$	$97.52\pm 2.51$	$97.85\pm 1.03$	$98.13\pm 0.99$	$98.30\pm 0.79$
Precision	$96.28\pm 3.27$	$97.01\pm 2.89$	$97.18\pm 2.75$	$97.69\pm 1.17$	$97.70\pm 1.25$	$98.00\pm 1.14$
Specificity	$99.39\pm 0.57$	$99.49\pm 0.48$	$99.39\pm 0.81$	$99.43\pm 0.28$	$99.35\pm 0.34$	$99.38\pm 0.31$
Recall	$96.71\pm 3.00$	$97.27\pm 2.78$	$97.67\pm 2.46$	$97.74\pm 1.03$	$97.92\pm 1.15$	$98.06\pm 0.90$
F1-score	$96.48\pm 3.14$	$97.13\pm 2.84$	$97.41\pm 2.55$	$97.71\pm 1.09$	$97.80\pm 1.18$	$98.03\pm 1.01$
Feature_2 (2 classes)						
Accuracy	$96.51\pm 2.63$	$97.41\pm 2.37$	$97.81\pm 2.14$	$98.38\pm 0.76$	$98.72\pm 0.64$	$98.74\pm 0.81$
Precision	$96.28\pm 2.73$	$97.08\pm 2.52$	$97.40\pm 2.47$	$98.22\pm 0.88$	$98.56\pm 0.71$	$98.60\pm 0.86$
Specificity	$96.28\pm 2.73$	$97.08\pm 2.52$	$97.40\pm 2.47$	$98.22\pm 0.88$	$98.56\pm 0.71$	$98.60\pm 0.86$
Recall	$96.42\pm 2.61$	$97.22\pm 2.42$	$97.63\pm 2.24$	$98.34\pm 0.78$	$98.58\pm 0.67$	$98.64\pm 0.83$
F1-score	$96.35\pm 2.67$	$97.15\pm 2.47$	$97.51\pm 2.35$	$98.28\pm 0.83$	$98.57\pm 0.69$	$98.62\pm 0.84$
Feature_2 (multiple classes)						
Accuracy	$96.18\pm 3.44$	$97.11\pm 2.79$	$97.48\pm 2.77$	$97.72\pm 1.14$	$98.05\pm 1.06$	$98.30\pm 0.86$
Precision	$96.12\pm 3.38$	$97.02\pm 2.84$	$97.13\pm 2.91$	$97.58\pm 1.21$	$97.67\pm 1.30$	$98.00\pm 1.10$
Specificity	$99.36\pm 0.61$	$99.49\pm 0.48$	$99.40\pm 0.78$	$99.39\pm 0.31$	$99.33\pm 0.37$	$99.38\pm 0.32$
Recall	$96.61\pm 3.06$	$97.25\pm 2.79$	$97.50\pm 2.68$	$97.62\pm 1.12$	$97.83\pm 1.20$	$98.05\pm 1.06$
F1-score	$96.35\pm 3.23$	$97.14\pm 2.82$	$97.31\pm 2.76$	$97.60\pm 1.16$	$97.74\pm 1.25$	$98.02\pm 1.06$
TPRO-NET (2 classes)						
Accuracy	$97.87\pm 1.89$	$98.08\pm 1.83$	$98.33\pm 1.55$	$98.86\pm 0.57$	$98.97\pm 0.49$	$98.93\pm 0.69$
Precision	$97.73\pm 1.92$	$97.82\pm 1.95$	$98.02\pm 1.87$	$98.76\pm 0.67$	$98.83\pm 0.56$	$98.81\pm 0.76$
Specificity	$97.73\pm 1.92$	$97.82\pm 1.95$	$98.02\pm 1.87$	$98.76\pm 0.67$	$98.83\pm 0.56$	$98.81\pm 0.76$
Recall	$97.82\pm 1.90$	$97.96\pm 1.86$	$98.20\pm 1.63$	$98.82\pm 0.57$	$98.85\pm 0.55$	$98.84\pm 0.72$
F1-score	$97.78\pm 1.91$	$97.89\pm 1.90$	$98.11\pm 1.74$	$98.79\pm 0.62$	$98.84\pm 0.55$	$98.82\pm 0.74$
TPRO-NET (multiple classes)						
Accuracy	$97.63\pm 2.38$	$97.74\pm 2.26$	$97.88\pm 2.24$	$98.18\pm 0.97$	$98.37\pm 0.93$	$98.40\pm 0.80$
Precision	$97.51\pm 2.34$	$97.65\pm 2.28$	$97.61\pm 2.50$	$98.06\pm 1.03$	$98.03\pm 1.13$	$98.10\pm 1.15$
Specificity	$99.60\pm 0.41$	$99.60\pm 0.40$	$99.49\pm 0.68$	$99.51\pm 0.26$	$99.44\pm 0.32$	$99.42\pm 0.31$
Recall	$97.85\pm 2.09$	$97.86\pm 2.15$	$97.86\pm 2.24$	$98.08\pm 1.02$	$98.22\pm 1.05$	$98.18\pm 0.90$
F1-score	$97.67\pm 2.22$	$97.75\pm 2.22$	$97.73\pm 2.35$	$98.07\pm 1.02$	$98.12\pm 1.08$	$98.14\pm 1.03$

### Ablation study

In order to improve efficiency while maintaining similar performance to transformer encoders, we have made improvements to transformer encoders. To verify the effectiveness of the improved transformer encoder, we conducted ablation experiments. The ablation experiment was conducted using the original transformer encoder instead of the improved transformer encoder. In the tables related to the experimental results, "Original_Transformer" is used to represent the original transformer encoder^40^.

The results of subject-dependent experiments are shown in the red line of Figs. 5, 6, 7, and 8, as well as Table 6. These results demonstrate that the improved transformer encoder achieves performance comparable to the original transformer encoder and even surpasses it in certain cases. Additionally, we measured the model parameters (Params) and Floating Point Operations (FLOPs) of the original transformer encoder and the improved transformer encoder on TPRO-NET to compare efficiency. When utilizing the original transformer encoder, TPRO-NET has Params of 1.20M and FLOPs of 9.90M. However, when utilizing the improved transformer encoder, TPRO-NET has Params of 1.10M and FLOPs of 9.11M. The results indicate that the improved transformer encoder can enhance computational efficiency by reducing Params and FLOPs by 8.33% and 7.98%.

**Table 6:** The results of the ablation study ($\text{mean}\pm \text{standard deviation}$).

Category	DEAP			DREAMER		
	Valence	Arousal	Dominance	Valence	Arousal	Dominance
Original transformer (2 classes)						
Accuracy	$97.15\pm 2.21$	$97.58\pm 2.23$	$97.86\pm 1.99$	$98.26\pm 0.80$	$98.61\pm 0.69$	$98.69\pm 0.79$
Precision	$96.95\pm 2.26$	$97.25\pm 2.33$	$97.48\pm 2.31$	$98.11\pm 0.95$	$98.44\pm 0.77$	$98.54\pm 0.89$
Specificity	$96.95\pm 2.26$	$97.25\pm 2.33$	$97.48\pm 2.31$	$98.11\pm 0.95$	$98.44\pm 0.77$	$98.54\pm 0.89$
Recall	$97.10\pm 2.21$	$97.41\pm 2.29$	$97.74\pm 2.09$	$98.19\pm 0.81$	$98.45\pm 0.76$	$98.60\pm 0.77$
F1-score	$97.02\pm 2.23$	$97.33\pm 2.31$	$97.60\pm 2.19$	$98.15\pm 0.88$	$98.44\pm 0.76$	$98.57\pm 0.83$
Original transformer (multiple classes)						
Accuracy	$97.33\pm 2.58$	$97.36\pm 2.55$	$97.44\pm 2.53$	$97.71\pm 1.09$	$97.93\pm 1.19$	$98.08\pm 1.01$
Precision	$97.31\pm 2.51$	$97.20\pm 2.61$	$97.12\pm 2.66$	$97.60\pm 1.17$	$97.48\pm 1.46$	$97.89\pm 1.10$
Specificity	$99.55\pm 0.45$	$99.53\pm 0.46$	$99.40\pm 0.70$	$99.39\pm 0.30$	$99.29\pm 0.40$	$99.30\pm 0.39$
Recall	$97.57\pm 2.39$	$97.39\pm 2.61$	$97.49\pm 2.56$	$97.60\pm 1.09$	$97.62\pm 1.43$	$97.80\pm 1.31$
F1-score	$97.43\pm 2.45$	$97.29\pm 2.60$	$97.30\pm 2.57$	$97.60\pm 1.12$	$97.54\pm 1.42$	$97.84\pm 1.20$
TPRO-NET (2 classes)						
Accuracy	$97.87\pm 1.89$	$98.08\pm 1.83$	$98.33\pm 1.55$	$98.86\pm 0.57$	$98.97\pm 0.49$	$98.93\pm 0.69$
Precision	$97.73\pm 1.92$	$97.82\pm 1.95$	$98.02\pm 1.87$	$98.76\pm 0.67$	$98.83\pm 0.56$	$98.81\pm 0.76$
Specificity	$97.73\pm 1.92$	$97.82\pm 1.95$	$98.02\pm 1.87$	$98.76\pm 0.67$	$98.83\pm 0.56$	$98.81\pm 0.76$
Recall	$97.82\pm 1.90$	$97.96\pm 1.86$	$98.20\pm 1.63$	$98.82\pm 0.57$	$98.85\pm 0.55$	$98.84\pm 0.72$
F1-score	$97.78\pm 1.91$	$97.89\pm 1.90$	$98.11\pm 1.74$	$98.79\pm 0.62$	$98.84\pm 0.55$	$98.82\pm 0.74$
TPRO-NET (multiple classes)						
Accuracy	$97.63\pm 2.38$	$97.74\pm 2.26$	$97.88\pm 2.24$	$98.18\pm 0.97$	$98.37\pm 0.93$	$98.40\pm 0.80$
Precision	$97.51\pm 2.34$	$97.65\pm 2.28$	$97.61\pm 2.50$	$98.06\pm 1.03$	$98.03\pm 1.13$	$98.10\pm 1.15$
Specificity	$99.60\pm 0.41$	$99.60\pm 0.40$	$99.49\pm 0.68$	$99.51\pm 0.26$	$99.44\pm 0.32$	$99.42\pm 0.31$
Recall	$97.85\pm 2.09$	$97.86\pm 2.15$	$97.86\pm 2.24$	$98.08\pm 1.02$	$98.22\pm 1.05$	$98.18\pm 0.90$
F1-score	$97.67\pm 2.22$	$97.75\pm 2.22$	$97.73\pm 2.35$	$98.07\pm 1.02$	$98.12\pm 1.08$	$98.14\pm 1.03$

### Experiments across all dimensions

Compared to running the algorithm separately in three dimensions to obtain emotions, running the algorithm once can significantly enhance practicality. To obtain the labels, a binary classification approach (Section 5.1) is used to divide the Valence, Arousal, and Dominance dimensions into high and low categories. The VA model uses two dimensions, Valence and Arousal, to create four labels by combining high and low labels from each dimension. Similarly, the VAD model employs three dimensions of Valence, Arousal, and Dominance to generate eight different labels by combining the high and low labels of different dimensions.

The experimental results of the subject-dependent experiment and the comparison with other advanced methods are shown in Table 7. The symbol '-' signifies that the pertinent data is not reported in the references. The experimental results show that TPRO-NET performs better than the existing advanced methods.

**Table 7:** The TPRO-NET performance of EEG-based emotion recognition across multiple dimensions ($\text{mean}\pm \text{standard deviation}$).

Methods	DEAP		DREAMER	
	VA	VAD	VA	VAD
GLFANET^39^	92.92 ± 1.30	–	–	–
SS kNN^41^	–	–	92.58	–
Transformer^7^	95.50 ± 5.07	94.49 ± 5.18	96.42 ± 5.81	95.28 ± 3.41
TC-NET^7^	**97.74 ± 1.93**	96.95 ± 2.73	97.69 ± 2.67	96.52 ± 2.77
TPRO-NET (this work)	97.45 ± 2.43	**97.71 ± 2.19**	**98.45 ± 0.72**	**98.37 ± 0.75**

Best performing values are in bold.

### Model efficiency

The Params and FLOPs are used to measure the model efficiency. After the measurement, TPRO-NET has FLOPs of 9.11M and Params of 1.10M. Table 8 shows the results of the comparison with other advanced models in FLOPs and Params. Although the Params in TPRO-NET is not the smallest, the number of FLOPs is the smallest, which can significantly improve efficiency.

**Table 8:** Comparison with other advanced methods in efficiency.

Methods	Params	FLOPs
DSE-Mixer^30^	**0.32 M**	90 M
MTL-MSRN^1^	1.0 M	350 M
FBCCNN(SOTA)^42^	2.12 M	120 M
STILN^31^	0.59 M	67.27 M
TPRO-NET (ours)	1.10 M	**9.11 M**

Best performing values are in bold.

### Comparison with other advanced peer methods

We compare our method with other advanced methods using the accuracy and standard deviation of the three dimensions of Valence, Arousal, and Dominance. When conducting binary classification, TPRO-NET outperforms other advanced methods. Furthermore, TPRO-NET exhibits strong classification ability in multi-classification scenarios. Table 9 shows the comparison results of advanced algorithms on the DEAP dataset and the DREAMER datasets.

**Table 9:** Comparison with other advanced methods in performance ($\text{mean}\pm \text{standard deviation}$).

Methods	DEAP			DREAMER		
	Valence	Arousal	Dominance	Valence	Arousal	Dominance
Fractal-SNN^43^	68.36	74.64	78.50	69.84	69.61	78.50
GLFANet (2 classes)^39^	94.53 ± 1.02	94.91 ± 1.05	95.35 ± 0.90	94.57	94.82	94.51
MTCA-CapsNet (2 classes)^44^	97.24 ± 1.58	97.41 ± 1.47	98.35 ± 1.28	94.96 ± 3.60	95.54 ± 3.63	95.52 ± 3.78
NAS^45^	**97.94 ± 1.04**	97.74 ± 1.02	97.82 ± 1.20	96.62 ± 3.52	96.29 ± 3.82	96.61 ± 4.04
TPRO-NET (2 classes, ours)	$97.87\pm 1.89$	$\mathbf {98.08\pm 1.83}$	$\mathbf {98.33\pm 1.55}$	$\mathbf {98.86\pm 0.57}$	$\mathbf {98.97\pm 0.49}$	$\mathbf {98.93\pm 0.69}$
TPRO-NET (multiple classes, ours)	$97.63\pm 2.38$	$97.74\pm 2.26$	$97.88\pm 2.24$	$98.18\pm 0.97$	$98.37\pm 0.93$	$98.40\pm 0.80$

Best performing value is in bold.

## Discussion

In this section, we first summarize the experiments and then discuss some details of the experiment.

The experimental results in Section "Experiments results and analysis" lead to three conclusions:

- Firstly, using an improved transformer encoder can achieve similar or higher performance with higher efficiency compared to the original transformer encoder.
- Secondly, the simultaneous use of enhancing DE features and DE features is complementary and can improve the performance of TPRO-NET.
- Finally, TPRO-NET can recognize more emotions compared to the current advanced methods, reflecting subtle changes in emotion.

Based on these conclusions, TPRO-NET has potential for practical application.

Validation of emotion recognition algorithms based on EEG signals can be subject-dependent or subject-independent. Our article uses the former to verify the effectiveness of TPRO-NET. However, for the latter, when using known emotional data from subjects to predict the emotions of unknown subjects, TPRO-NET performed commonly. We speculate different individuals have different feelings towards the same induced material, which leads to different emotions. TPRO-NET is currently difficult to infer the emotions of unknown individuals using the emotional data of known individuals.

In Eq. 4, we use a simple logarithmic transformation to enhance the differential entropy feature. The results of the experiments in Section "Influence of different types of features on the results" demonstrate that utilizing both features concurrently can enhance performance. Moreover, we utilized the exponential function with a base of e and the linear transformation of 1-M to improve the differential entropy feature. The former result in a decrease in TPRO-NET's performance, while the latter enhance performance but also has the potential to cause training crashes at unpredictable times. Although the use of simple transformations may decrease the computational efficiency of the algorithm, the performance of the algorithm is constantly improving with the development of deep neural networks. It is worth studying how to improve performance by using simple linear and nonlinear transformations. As the reasonable utilization of these transformations is a complex topic, further research will be conducted in the future.

## Conclusion

In this article, we propose an EEG signal-based emotion recognition algorithm, TPRO-NET, which uses differential entropy features and enhanced differential entropy features as inputs and obtains the final emotion category through convolutional layers and improved transformer encoders. Experiments on the DEAP and the DREAMER datasets demonstrate that TPRO-NET achieves state-of-the-art results in completing complex multiple emotion classification tasks, distinguishing subtle emotional changes. In addition, the experiments demonstrate the effectiveness of the differential entropy feature enhancement method and the improved transformer encoder for complex sentiment classification tasks. We plan to investigate the issue of TPRO-NET's generally average performance in subject-independent experiments, the impact of simple transformations on existing features and the practical application of TPRO-NET.

## Data Availability

The DEAP dataset and the DREAMER dataset are both publicly available. They can be accessed from https://www.eecs.qmul.ac.uk/mmv/datasets/deap/ and https://zenodo.org/records/546113, respectively.

## Code availability

To enhance academic mutual assistance and benefit, the source code of this work can be shared with the requester for non-commercial, pure scientific research upon reliable inquiry.
